# An important diagnostic marker of acute myocardial infarction patients: Plasma miRNA133 levels

**DOI:** 10.1097/MD.0000000000038781

**Published:** 2024-07-19

**Authors:** Xiaona Cai, Jialin Shi, Yangmiao Xu, Liying Fu, Xiuming Feng, Ruifang Zhao

**Affiliations:** a Department of Cardiology, Shaoxing People’s Hospital, Shaoxing, Zhejiang, China; b School of Medicine, ShaoXing University, Shaoxing, Zhejiang, China.

**Keywords:** acute myocardial infarction, diagnostic marker, MiRNA133, specificity

## Abstract

The objective of this study was to explore changes in miRNA133 levels as a basis for clinical diagnostic markers in patients with acute myocardial infarction (AMI). A total of 100 chest pain patient cases admitted to a hospital from June 2021 to December 2022 were used. The study involved the selection of 50 patients: 25 patients with unstable undetermined heart pain and 25 healthy subjects were included in the control group of 50 patients with non-AMI patients. Meanwhile, 50 patients with AMI were designated as the experimental group. Changes in miRNA133 levels in patients’ plasma were analyzed for expression using quantitative fluorescence analysis. When the serum TPI, plasma NT-ProBNP, glycosylated hemoglobin, and plasma D-dimer index values were compared between the control and experimental groups, there was a statistically significant difference (*P* < .05). mi-RNA-133 had a mean plasma level value of 2.60 ± 1.01, the mean level value of mi-RNA-133 in patients with non-AMI was 1.34 ± 1.18, and the patients in the AMI group showed significantly high values of the mean plasma level of mi-RNA-133. The relative expression level value of cTnl in patients with AMI was 10.84 ± 12.64. Of the specificity and sensitivity diagnostics, mi-RNA-133 had the best diagnostic effect. The area under mi-RNA-133 in the regression curve was 95.4%, the specificity of the whole combination of indicators was 89.4% and the sensitivity was 100%. Finally, the correlation between mi-RNA-133 and white blood cell count (WBC) and TG was statistically significant (*P* < .05). In conclusion, changes in the level of mi-RNA-133 may be an important marker for diagnosing the status of patients with AMI, while a faster and more accurate method will emerge along with the improvement of the detection technology, and at the same time, due to the variability of the study cases and other limitations, further research will be carried out subsequently.

## 1. Introduction

Acute myocardial infarction (AMI) is a dangerous kind of heart illness that is typically brought on by an abrupt blockage of the coronary arteries, which causes myocardial necrosis and ischemia.^[1]^ For prompt treatment and a better prognosis for the patient, an early diagnosis of AMI is essential. However, there are limitations in current AMI diagnostic methods, including electrocardiography and biomarker testing, which may not be sensitive and specific enough for early diagnosis and prognostic assessment.^[2]^ As an endogenous non-coding single-stranded RNA structure, mi-RNA is able to regulate a variety of physiological processes, and in the process, it can also promote the degradation and transcription of messenger RNAs, which in turn regulates the expression of other RNAs.^[3]^ At the same time, miRNAs will combine with complex protein complexes during the cycling process to reach a relatively stable form in plasma, which is easy to detect and express.^[4]^ In the current study some miRNAs in AMI patients can be released through myocardial infarction (MI) cells, which in turn elevates the content of miRNAs in patients.^[5,6]^ The content of specific miRNAs can detect the degree of cardiac damage in current patients, so miRNAs are of interest as a marker diagnostic for AMI.^[7–9]^ Meanwhile, in the current study, the detection of creatine kinase (CK) and cardiac troponin (CTn) is also a more advanced diagnostic judgment standard for AMI. However, CTn and Ck have limitations in research and detection, and the levels of the 2 indicators are also abnormal in other cardiac diseases, so the research and analysis of AMI requires the cooperation of multiple indicators and variables for diagnosis.^[10]^ Therefore, this study aimed at the limitations of the current AMI diagnosis, explored the correlation between the expression of mi-RNA-133 content and AMI diagnosis, and explored the potential value of the current research direction, so as to point out a new research direction for the subsequent diagnosis of AMI.

## 2. Materials and methods

### 2.1. Research objects

This study was approved by the Ethics Committee of Shaoxing People Hospital. For the study, 100 cases of patients who were admitted hospital between June 2021 and December 2022 were chosen, with 50 patients with AMI serving as the experimental group and 50 HC (Health Control) patients as the control group.^[11]^ The HC group was a physically healthy population; none of them were diagnosed with cardiovascular disease. The diagnostic criteria for AMI patients were selected from the European Heart Association fourth diagnostic criteria definition for AMI.^[12]^ Before blood samples were taken for the study, patients or their relatives are aware of the circumstances and the goal of the experiment. Informed consent was obtained from the patients.

### 2.2. Inclusion criteria

AMI group: clinical manifestations include the presence of chest pain or chest discomfort, which may radiate to the neck, jaw, back, shoulder, or arm, accompanied by systemic or gastrointestinal symptoms, cardiac arrhythmia, blood pressure drop, and heart failure. The creatine kinase isoenzyme (CK-MB) index abnormalities, CTnT and I elevation, ST-segment elevation or non-ST-segment elevation, and other abnormalities are examples of ECG appearances.

Control group: patients with normal electrocardiogram, normal CTn indexes, the rest of the laboratory blood markers are normal (blood markers within normal reference range), and no obvious abnormalities in clinical examination. Clinical examination of the healthy subject population.

### 2.3. Exclusion criteria

Patients with comorbid other diseases of the cardiovascular system, presence of previous coronary heart disease, cardiac hypertrophic disease and valvular heart disease, Patients with a history of MI were excluded from the trial; history of thyroid disease, impaired liver function, impaired renal function, autoimmune diseases such as systemic lupus erythematosus, and malignant neoplastic diseases were excluded; and history of traumatic injury to the skeletal muscle. The ethics committee gave its approval to the project.

### 2.4. Baseline information

The basic clinical information data of all subjects were collected through the hospital case data system, which included the patients’ age, gender, BMI index, history of hypertension, history of diabetes, and blood tests on the collected subjects included serum cTnI, plasma NT-ProBNP, glycosylated hemoglobin, plasma D-dimer, left ventricular ejection fraction (LVEF) on cardiac ultrasound, troponin cTnI, red blood cell count (RBC), white blood cell count (WBC), platelet count (PLT), left ventricular diastolic end-stage internal diameter (LVDd), CK-MB, triglyceride TG, total cholesterol TC, and cholesterol LDLC.

### 2.5. Materials and reagents

Pipette gun (Eppendorf), ultra-low temperature refrigerator −20°C (Zhongke Meiling Company) and −80°C (Zhongke Meiling Company), ice machine (SANYO), high-speed centrifuge (Ruibang Xingye), DEPC (Amresco), agarose, M5HiClearDL2000DNAmarker, mi-RNA first-strand cDNA synthesis (Shanghai Sangong), mi-RNA fluorescence quantitative PCR kit (Shanghai Sangong), high-speed freezing centrifuge (Shanghai Lixin Scientific Instrument Co., Ltd.), vortex mixer (Haimen Qilinbel Instrument Manufacturing Co., Ltd.), microspectrophotometer (Hangzhou Aosheng), gel imaging system (Shanghai Tannen), BG-Piwer600i routine electrophoresis instrument (Beijing Baijing Biotechnology Co. Ltd.), electrophoresis tank (Beijing Liuyi Instrument Factory), PCR instrument (Bio-Rad), chloroform, isopropanol, anhydrous ethanol (Beijing Chemical Factory), enzyme-free EP tubes (Axygen), enzyme-free pipette tips (Axygen), RNase-free water (Bio-Rad), cel-miRNA133 standard (Guangzhou Ruibo Biotechnology Co. product (Guangzhou Ruibo Biotechnology), Bulge-LoopTMmiRNA-133 Primer (Guangzhou Ruibo Biotechnology).

### 2.6. Method of use

#### 2.6.1. Sample collection

A PAXgene Blood RNA tube was used to collect approximately 2 mL of patient venous blood. These samples were left at room temperature for 2 hours and subsequently transferred to a −20°C refrigerator for 24 hours. Upon completion of this stage, the samples were transferred to a deep freezer at −80°C for long-term storage.

#### 2.6.2. Mi-RNA screening method

Remove the clinical sample from the refrigerator at −80°C and warm it up to room temperature. The sample was subjected to centrifugation operation, the centrifugation time was 10 minutes speed of about 12000pm. Selected the upper layer of the sample in the clear sample value EP tube, add TRIzrl about 1 mL in the tube, mix thoroughly. After standing for about 10 minutes add cel-miRNA133 standard, mix thoroughly and stand for 5 minutes. Add chloroform about 200 µL and stand for 5 minutes. Centrifuge for about 10 to 20 minutes. Transfer the upper phase to a fresh EP tube, fully combine with isopropyl alcohol, and store at −20°C overnight for use the following day. When centrifuging the sample, be careful not to touch the lower layer of precipitate. Add 75% ethanol to the EP tube to clean the precipitate of the lower phase, and centrifuge again for about 10 minutes after manual shaking. Repeat step (9) twice after removing the upper layer of the clear sample. After all the upper layer was aspirated, the sample was dried at room temperature for about 15 minutes. The precipitate was finally dissolved with roughly 50 µL of RNase-free water and stored in a refrigerator at −80°C as a reserve.

#### 2.6.3. RNA transcription process

The reverse transcription process was systematically configured according to Table 1

**Table 1:** Transcription configuration system.

Template	Dosage
mi-RNA RTase mixture	2 µL
RNase-free Water	15 µL
RTase Mix	2 µL
mi-RNA RT Enzyme mix	2 µL
2 × mi-RNA RT Solution mixI	10 µL

The RNA was configured for reverse transcription according to the system in Table 1, and after all systems were mixed well, centrifugation was performed.^[13]^ The reverse transcription process was carried out on a PCR amplifier with temperature and time control of reverse transcription at 42°C for 60 minutes.^[14, 15]^ Afterwards, reverse transcription was performed at 85°C for 5 minutes to inactivate the transcriptase and end the reverse transcription process. The transcribed cDNA was stored at −80°C.

#### 2.6.4. Quantitative fluorescence measurements

The fluorescence reaction system configuration criteria are shown in Table 2.

**Table 2:** Configuration of fluorescence reaction system.

Standard material	Dosage
2XmiRNA qPCR MasterMix	10 µL
Forward Primer	0.5 µL
Reverse Primer	0.5 µL
Complementary DNA	2 µL
Bulge-Loop^TM^miRNA-133Primer	1 µL
RNase-free H2O	Increase to 20 µL

The system configurations in Table 2 were mixed and then centrifuged, the fluorescence reaction was carried out in a CA detection system, the reaction was incubated at 95°C for 10 minutes, after which it was left at 95°C for 2 seconds for the pre-denaturation phase, 60°C for 20 seconds for the denaturation phase, and 60°C for 10 seconds for the elongation phase, respectively, with the denaturation and elongation were cycled 40 times, respectively.^[16]^ Afterwards, the sample samples were heated from 60°C to 95°C to obtain a melting curve, and mi-RNA expression was assessed using the $\;2^{-\Delta \Delta Ct}$ (ΔCt= CtmiRN = Ctcel-miR-39) method using cel-mi-RNA-39 as a control sample.

### 2.7. Methods of statistical analysis

With SPSS 26.0, the currently acquired data were statistically examined. All tested data samples were analyzed using mean±standard error using the trend of normal distribution reflecting the current changes in the distribution of continuous variables.^[17,18]^ Multifactorial group differences Multivariate ANOVA was selected for testing differences between 3 and more groups of components. Wilcoxon signed-rank test was used to compare differences between 2 groups of components. Mi-RNA expression analysis of AMI concern blood samples and normal control group blood samples was analyzed using the $\;2^{-\Delta \Delta Ct}$ method.^[19]^ The correlation of mi-RNA-133 with cTnl, CK-MB, and WBC was analyzed using Pearson test, and logistic regression analyzed the independent predictive ability of mi-RNA-133 for AMO. ROC curves were used to assess the diagnostic efficacy of mi-RNA as a diagnosis of AMI. The diagnostic ability judgment criteria corresponded to values of 0.5 to 0.7 for low diagnostic efficacy, 0.7 to 0.85 for medium diagnostic efficacy, and 0.85 to 1.0 for high diagnostic efficacy.^[20]^ The BMI index judgment criteria were selected as 18.5 to 24, and patients with >24 were diagnosed as obese, while those with <18.5 were diagnosed as emaciated. All statistical testing data used 2-tailed (*P* < .05).

## 3. Results

### 3.1. Comparison of clinical baseline data

Inclusion criteria in the study June 2021 to December 2022 number of cases 100, of which 25 UAP and 25 HC in the control group and 50 AMI patients were included in the experimental group. Table 3 inclusion criteria were used to select 50 AMI patients for the experimental group and, as a control experiment, 25 UAP and 25 healthy individuals for the control group. Table 3 shows that there were statistically significant (*P* < .05) variations in the patients’ WBC, RBC, total cholesterol (TC), low-density lipoprotein cholesterol (LDLC) and TG levels. When comparing gender, age, past history of hypertension, past history of diabetes mellitus, BMI index, and PLT values in Table 3, there was no statistically significant difference.

**Table 3:** Comparison of basic baseline data.

Variables	UAP (n = 25)	HC (n = 25)	AMI (n = 50)	*P* value
Age (yr)	60.24 ± 11.25	58.47 ± 10.25	61.56 ± 10.25	.56
Gender (woman)	10	12	13	.14
WBC (10^9^/L)	6.01 ± 0.94	6.10 ± 1.45	10.14 ± 4.16	<.05
Hypertension (n)	15	14	26	.51
Diabetes mellitus (n)	16	19	30	.47
BMI (18.5–24)	20	18	13	.61
RBC (10^12^/L)	5.01 ± 0.46	4.36 ± 0.41	4.84 ± 0.50	<.05
PLT (10^9^/L)	225.12 ± 50.35	234 ± 70.64	222.56 ± 60.12	.54
TC (mmol/L)	4.65 ± 0.74	3.48 ± 1.32	4.87 ± 1.12	<.05
TG (mmol/L)	0.99 ± 0.24	1.84 ± 1.43	1.58 ± 0.83	<.05
LDLC (mmol/L)	2.36	3.12	3.57	<.05

HC = health control, PLT = platelet count, RBC = red blood cell count, WBC = white blood cell count.

### 3.2. Clinical baseline comparison between acute and non-acute infarction groups

When comparing the WBC, LDLC and mi-RNA-133 level contrast between the N-AMI control group and the AMI experimental group, as indicated in Table 4, a statistically significant difference (*P* < .05) was found. When comparing the N-AMI control group to the AMI experimental group, there were no statistically significant differences in WBC, RBC, TC, TG, gender, age, history of previous hypertension, history of prior diabetic mellitus, BMI indices, and PLT values.

**Table 4:** Clinical baseline comparison between acute myocardial infarction group and non-acute myocardial infarction group.

Variables	N-MI(n = 50)	AMI(n = 50)	*P* value
Age (yr)	59.47 ± 10.25	61.26 ± 11.25	.43
Gender (woman)	22	13	.08
WBC (10^9^/L)	6.01 ± 1.15	10.18 ± 4.19	<.05
Hypertension (n)	29	26	.51
Diabetes mellitus(n)	35	30	.47
BMI (18.5–24)	39	13	.61
RBC (10^12^/L)	4.36 ± 0.41	4.42 ± 0.51	.81
PLT (10^9^/L)	231.24 ± 68.64	222.54 ± 58.57	.54
TC (mmol/L)	1.48 ± 1.01	1.47 ± 0.89	.64
TG (mmol/L)	1.65 ± 1.12	1.58 ± 0.83	.57
mi-RNA-133	0.08 ± 0.13	1.87 ± 1.54	<.05

PLT = platelet count, RBC = red blood cell count, WBC = white blood cell count.

### 3.3. Comparison of cTnl and mi-RNA-133 index levels in patients

After comparing the N-MI control group with the AMI experimental group, as shown in Table 5, there is a statistically significant difference (*P* < .05) in comparing the cTnl index level and mi-RNA-133 level of patients in the first blood collection. There was a statistically significant difference (*P* < .05) in the comparison of cTnl index levels and mi-RNA-133 levels in patients with the second blood collection.

**Table 5:** Changes in cTnl index levels after secondary blood collection in patients.

Patient group	Point of time	mi-RNA-133	cTnI (ng/mL)	*P* value
AMI (n = 50)	First blood collection (initial diagnosis)	1.87 ± 1.54	0.12	<.05
AMI (n = 50)	Second blood collection (24 h after diagnosis)	1.95 ± 1.35	1.25	<.05
N-MI (n = 50)	First blood collection (initial diagnosis)	0.08 ± 0.13	0.02	<.05
N-MI (n = 50)	Second blood collection (24 h after diagnosis)	0.06 ± 0.11	0.03	<.05

### 3.4. Comparison of the levels of cardiovascular indices between the control and experimental groups

The comparison of cardiovascular indices between AMI and N-AMI, as indicated in Table 6, revealed a statistically significant difference (*P* < .05), comprehensive serum troponin cTnl from patients in the experimental and control groups on secondary blood collection, plasma NT-ProBNP, glycosylated hemoglobin, and plasma D-dimer index values. In general, AMI patients had higher values of numerous markers than N-AMI patients. The experimental group and the control group did not exhibit any statistically significant differences in the values of LVDd or LVEF on cardiac ultrasonography.

**Table 6:** Comparison of cardiovascular indices between AMI and N-AMI.

Variables	N-AMI (n = 50)	AMI (n = 50)	*P* value
TPI (µg/L)	0.24 ± 0.34	0.54 ± 0.25	<.05
NT-ProBNP (pg/mL)	118.15 ± 12.53	126.35 ± 14.25	<.05
HbA1c (%)	6.01 ± 1.15	10.18 ± 4.19	<.05
D-Dimer (mg/L)	0.26 ± 0.64	0.67 ± 0.45	<.05
LVEF (%)	64.35 ± 15.36	80.78 ± 12.65	.21
LVDd (mm)	46.48 ± 9.65	64.46 ± 11.35	.25

LVDd = left ventricular diastolic end-stage internal diameter, LVEF = left ventricular ejection fraction.

### 3.5. Comparison of mi-RNA-133 level expression in the combined results of 2 sampled plasmas

**Figure 1.**
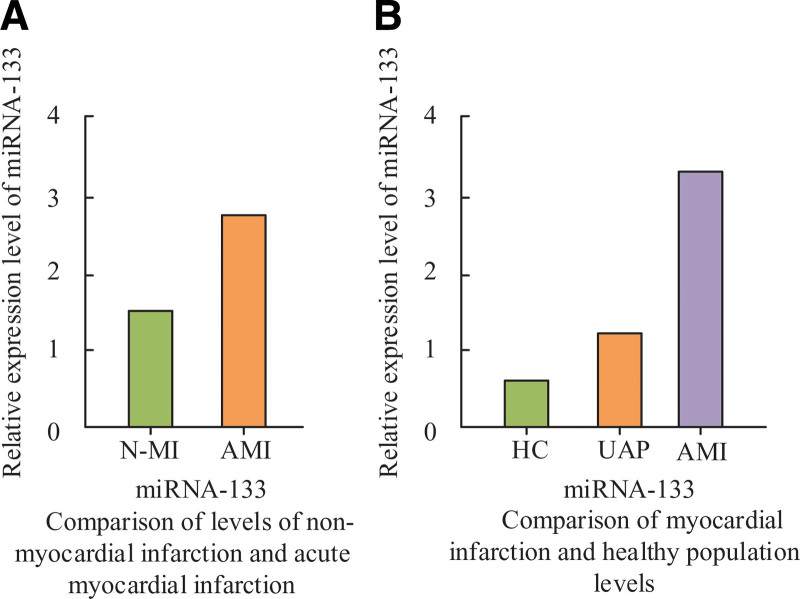
Comparison of mi-RNA-133 levels in plasma between control group and experimental group.

In Figure 1A, mi-RNA-133 horizontal expression in plasma differed in the index levels in AMI patients and N-AMI patients. In patients with AMI and non-AMI, the mean level value of mi-RNA-133 was 2.60 ± 1.01 in patients with AMI and 1.34 ± 1.18 in patients with non-AMI. In the figure, patients in the AMI group had significantly higher mean level values of mi-RNA-133 in plasma. The mi-RNA-133 levels averaged 3.65 ± 1.42, 1.13 ± 0.96 and 0.78 ± 0.35 in patients with AMI and unstable undetermined cardiac pain and in healthy subjects, respectively, as shown in Figure 1B. It was evident from the figure that when the 3 groups were compared, the relative amount of mi-RNA-133 expression rose with time. The relative level of expression in unstable UAP and HC was compared with a significant increase in mi-RNA-133 levels in patients.

### 3.6. Comparison of differential expression of cTnl and CK-MB levels in peripheral blood of patients

**Figure 2.**
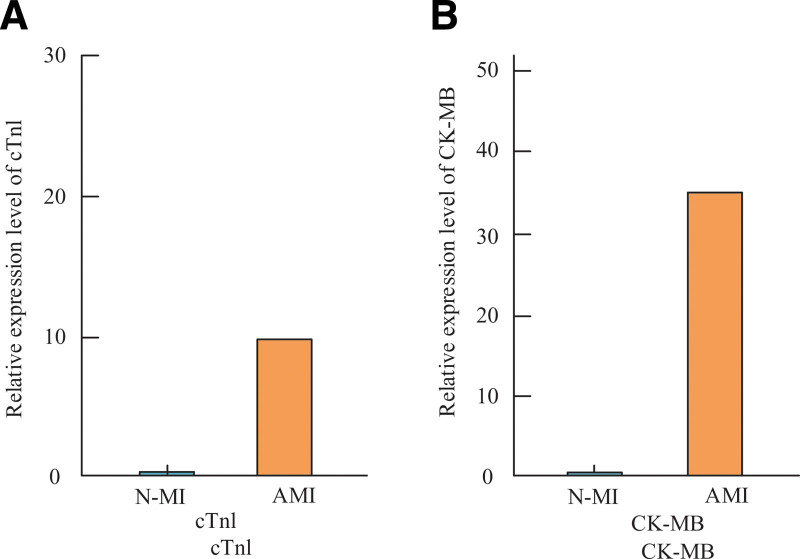
Comparison of peripheral blood parameters in patients.

The peripheral blood data of the patients were queried through the hospital medical record system, from which the combined peripheral blood CK-MB and cTnI levels were also retrieved from the system on 2 occasions of blood collection. Furthermore, Figure 2 clearly shows that AMI patients’ peripheral blood had considerably greater relative expression levels of cTnl and CK-MB than did N-AMI patients. The study found that there was a statistically significant difference (*P* < .05) in the relative expression level of cTnl between AMI patients (10.84 ± 12.64) and N-AMI patients (0.25 ± 0.11). CK-MB was 35.35 ± 39.58 in AMI patients and 1.16 ± 1.22 in N-AMI patients (*P* < .05). CK-MB relative expression level value in AMI patients was 1.05 ± 1.12 (*P* < .05).

### 3.7. Comparative analysis of mi-RNA-133 sensitivity and specificity

**Figure 3.**
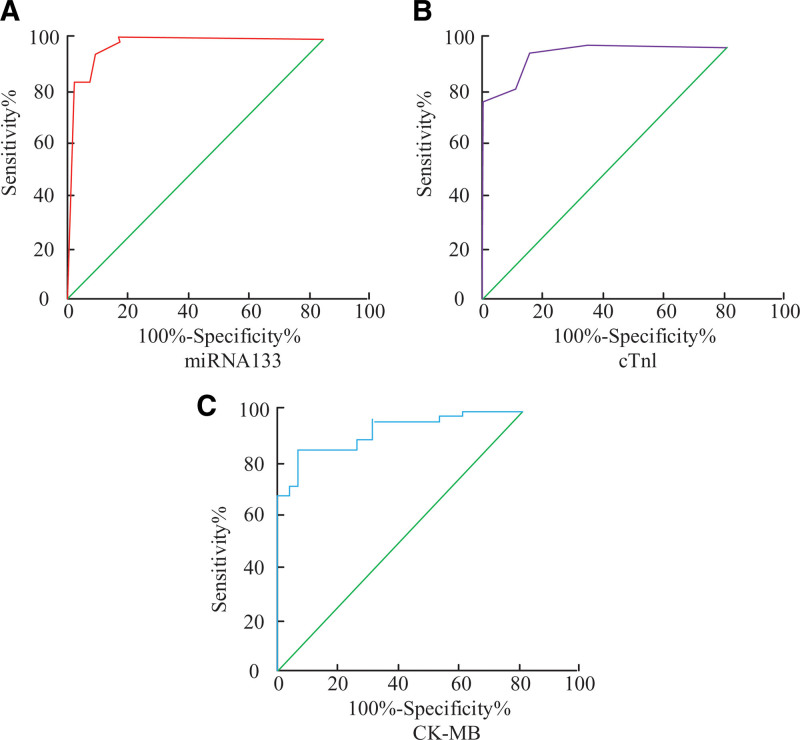
ROC curves of cTnl and mi-RNA-133 in the subjects.

Plotting the subject patients’ ROC curves allowed for an analysis of the impact of mi-RNA133 on AMI patients as well as their present diagnostic status. The diagnostic ability of each index is indicated by the size of the area under the curve, and the larger the area, the better the diagnostic ability and diagnostic effect. As shown in Figure 3 among the 3 indicators, the diagnostic ability and diagnostic effect of mi-RNA133 were the best, and the areas under the curves of mi-RNA133, CK-MB and cTnl were 0.956 (95.6%), 0.839 (83.9%), and 0.897 (89.7%), respectively. The sensitivity and specificity of identifying AMI were 87.6% and 94.7%, respectively, when the level of mi-RNA133 in AMI patients was 36.5%. In individuals with AMI, a CK-MB level of 32.2% resulted in 79.5% sensitivity and 81.5% specificity in the diagnosis of AMI. The sensitivity and specificity of identifying AMI were 85.7% and 89.9%, respectively, when the cTnl level of AMI patients was 31.6%. As demonstrated in the image, mi-RNA133 has superior specificity and sensitivity for AMI patients than the CK-MB and cTnl indexes, and it also improves the diagnosis of AMI patients.

### 3.8. Logistic regression curve analysis combined with diagnostic modeling

**Figure 4.**
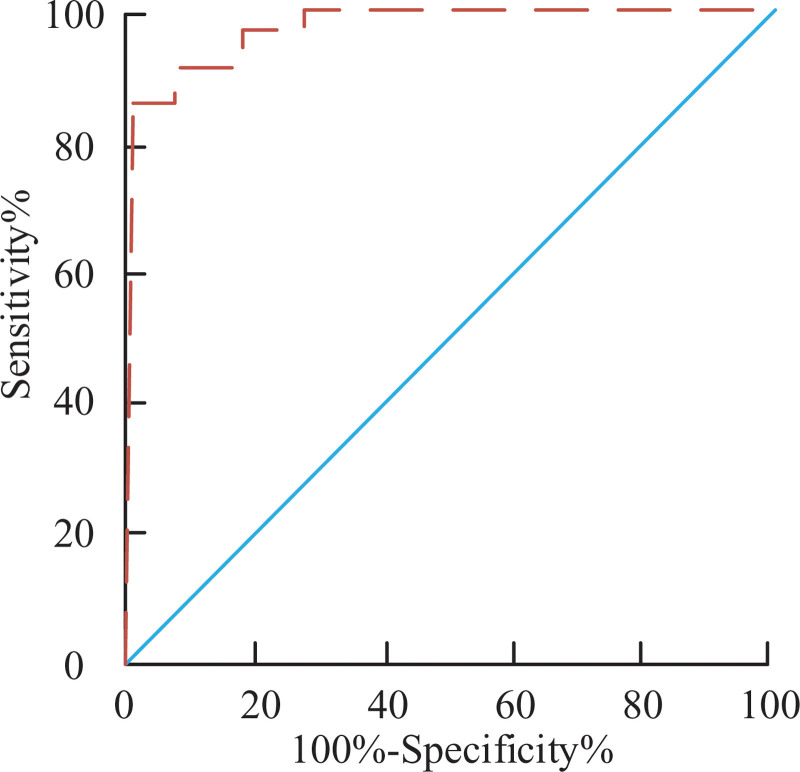
Combination logistic regression curve.

The expression diagnostic results of the indicators of mi-RNA133 and the expression results of the indicators of CK-MB and cTnl were analyzed by logistic regression curves to build a joint model of the combined indicators. The size of the area under the curve indicates the specificity and sensitivity of the curve. Figure 4 shows a sensitivity of 100%, an area under the curve of 95.4%, and a specificity of 89.4% for the entire set of indicators. The new regression curve value of 0.251 is obtained by evaluating the fitting calibration of the combined indicators, which indicates that the whole model is well fitted, and the formula of the whole model is shown in equation (1).

$$\begin{array}{l} \begin{array}{lll} & Logit(\;p)=3.82-5.663\times mi-RNA-133\; & -0.195\times EXP_{CK-MB}-6.89\times EXP_{cTnl}\; \end{array} \end{array}$$ (1)

To further test and analyze the regression equation, a randomized trial sample of 40 cases, including 20 patients with AMI and 20 patients with N-AMI were blinded and *P* > 1.0579 was taken as the test criterion, and the final calculation yielded a sensitivity of 94.6% and a specificity of 87.02%.

### 3.9. Correlation analysis of mi-RNA-133

Correlation analysis using spearman correlation rank was performed, and the correlation between mi-RNA-133 and each index obtained from AMI patients was shown in Table 7. mi-RNA-133 showed positive correlation with WBC, RBC, TC, and TG, with statistically significant correlations with mi-RNA-133 and WBC, and TG (*P* < .05). With regard to RBC, PLT, and TC, there was no statistically significant link. A negative correlation (r = −0.08) was seen between mi-RNA-133 and PLT.

**Table 7:** Correlation analysis of mi-RNA-133.

Variables	mi-RNA-133	
	r	*P* value
WBC (10^9^/L)	0.52	<.05
RBC (10^12^/L)	0.07	.52
PLT (10^9^/L)	−0.08	.42
TC (mmol/L)	0.21	.02
TG (mmol/L)	0.25	<.05
mi-RNA-133	−0.52	<.05

PLT = platelet count, RBC = red blood cell count, WBC = white blood cell count.

## 4. Discussion

AMI, one of the current cardiovascular diseases with high morbidity and mortality, is a serious cardiac condition that occurs when a part of the heart suffers damage or death of cardiac muscle cells due to a sudden interruption in blood supply.^[21]^ One of the main causes of adult mortality worldwide is AMI.^[22]^ Some of the current indicators commonly used in clinical practice can be used as a marker diagnostic of AMI status such as troponin, which is elevated to mark the occurrence of AMI, but at the same time, other cardiovascular diseases can bring about elevation of troponin and decrease the diagnostic effectiveness of AMI.^[23]^ Also, the elevation of troponin levels may be delayed for several hours and is not specific. Therefore, the search for earlier and more accurate biomarkers is important to improve the diagnosis and treatment of AMI.^[24]^ MicroRNAs, a class of non-coding small molecule RNAs, have become increasingly significant in the pathophysiology of cardiovascular disorders in recent times. MiRNAs affect a range of biological processes, by modulating gene expression. miRNA133, a mi-RNA extensively expressed in the heart, has been demonstrated to have a crucial role in the proliferation and death of cardiomyocytes. Several studies have shown that the expression level of miRNA133 fluctuates after MI, which suggests that miRNA133 may be employed as a biomarker for the early identification of AMI.^[25]^ The study of changes in the level of miRNA133 in plasma of AMI patients provides a new direction for a deeper understanding of the molecular mechanism of AMI. By analyzing the expression level of miRNA133 in plasma, it may not only help in the early diagnosis of AMI, but may also reveal the extent of myocardial injury, thus providing important information for the treatment and prognosis assessment of patients. In addition, changes in miRNA133 levels may also be associated with different clinical types and pathophysiological states of AMI, which is potentially valuable for the development of individualized therapy.

MiRNA133, encoded as a gene on human chromosome 6, is widely present in muscle tissues and myocytes, and at the same time is able to participate in the process of apoptosis and decay of several physiological cells and physiological tissues, as well as in the differentiation of cardiac myocytes and the regeneration of cardiovascular cells.^[26]^ Numerous investigations have demonstrated the connection between miRNA133 expression and the diagnosis of several cardiovascular conditions, including AMI, hypertension, coronary heart disease, etc. In the current study abnormal miRNA133 expression was present in some AMI patients, leading to a significant increase in their plasma miRNA133 levels, and miRNA133 was also found to be involved in blood circulation in the coronary arteries of some patients with MI.^[27]^ In some studies, it has been shown that miRNA133 levels are able to play a regulatory role in cancer, while mi-RNA expression in plasma has been suggested to be a marker for diagnostic judgment in AMI.^[28]^ With the depth of current research, it can be demonstrated that miRNAs have great potential in AMI and can be tested and judged as the current clinical landmark diagnostic substances.^[29]^

Consequently, it was discovered that miRNA133 can be utilized as a current signature diagnostic for AMI in a study on the gene.^[30]^ When comparing the BMI index, PLT levels, history of prior diabetes, history of prior hypertension, history of age, and gender among AMI patients, no statistically significant differences were found. When WBC and mi-RNA-133 levels were compared between the experimental and control groups, there was a statistically significant difference (*P* < .05). There was a statistically significant difference (*P* < .05) in comparing WBC, RBC, total cholesterol (TC), low-density lipoprotein cholesterol (LDLC), and triglyceride content (TG) in the experimental and control group of patients. The levels of glycosylated hemoglobin, plasma D-dimer, serum, and plasma in the experimental and control groups were statistically significantly different (*P* < .05). There was no statistically significant difference between the values of LVDd and LVEF on cardiac ultrasonography. It can be seen that there will be differences between AMI patients and N-AMI patients in some indexes, which can determine whether AMI condition occurs or not, which has positive significance in determining AMI patients. There was a statistically significant difference (*P* < .05) in the comparison of cTnl index levels and mi-RNA-133 levels in patients with the first blood collection. There was no statistically significant difference in the comparison of cTnl index levels and mi-RNA-133 levels in the second blood collection. This indicates that at the beginning of the diagnostic period the cTnl and mi-RNA-133 levels of the patients were at relatively low values, and 3 hours after the diagnostic period, the levels of the indicators in the patients increased significantly. The mean value of mi-RNA-133 levels in patients with AMI was 2.60 ± 1.01, and in patients with non-AMI 1.34 ± 1.18. The mi-RNA-133 values were significantly increased. mi-RNA-133 levels were significantly increased in AMI patients and unstable UAP and HC with mean levels of 3.65 ± 1.42, 1.13 ± 0.96和0.78 ± 0.35. The mean level of the patients was significantly higher, which indicates that the value of mi-RNA-133 level expression was increased in AMI patients, which also signifies the ability to determine whether the current patient has AMI or not by the value of mi-RNA-133 level. The hospital medical record system was utilized to query the patients’ peripheral blood data. Figure 2 illustrates that patients with AMI had considerably greater relative expression levels of cTnl and CK-MB in their peripheral blood compared to those with N-AMI. Similarly, the relative expression level value of CK-MB between the experimental and control groups was 1.16 ± 1.22. This indicates that the difference in the relative levels of CK-MB and cTnl can be used as a marker to determine AMI. The diagnostic ability and diagnostic effect of mi-RNA133 were the best in the ROC curve, and the areas under the curves of mi-RNA133, CK-MB and cTnl were 0.956 (95.6%), 0.839 (83.9%) and 0.897 (89.7%), respectively. This indicates that when comparing the diagnostic effects and markers of mi-RNA133, CK-MB and cTnl as the diagnostic effects and markers of AMI patients, the diagnostic effect of mi-RNA133 is better and can reflect the current patient disease condition.

In summary, alterations in mi-RNA133 content can serve as a diagnostic marker for AMI, and compared to other indicators, mi-RNA133 has superior specificity and difference. Therefore, the study of mi-RNA133 as a diagnostic status judgment of AMI has better practical value and significance. Since the sample size of the study is relatively small, more sample data will be analyzed in the future.

## Author contributions

**Conceptualization:** Xiaona Cai, Jialin Shi, Ruifang Zhao.

**Data curation:** Xiaona Cai, Jialin Shi, Yangmiao Xu, Xiuming Feng, Ruifang Zhao.

**Formal analysis:** Xiaona Cai, Jialin Shi, Yangmiao Xu, Liying Fu, Xiuming Feng, Ruifang Zhao.

**Investigation:** Xiaona Cai, Jialin Shi, Yangmiao Xu, Liying Fu, Xiuming Feng, Ruifang Zhao.

**Methodology:** Xiaona Cai, Jialin Shi, Liying Fu, Xiuming Feng, Ruifang Zhao.

**Supervision:** Liying Fu, Ruifang Zhao.

**Validation:** Yangmiao Xu.

**Visualization:** Jialin Shi.

**Writing – original draft:** Xiaona Cai.

**Writing – review & editing:** Xiaona Cai, Jialin Shi, Ruifang Zhao.
